# Atopic dermatitis, venous thromboembolism and cancer: a cohort analysis

**DOI:** 10.1007/s11239-025-03175-2

**Published:** 2025-09-04

**Authors:** Sissel Brandt Toft Sørensen, Cecilia H. Fuglsang, Erzsébet Horváth-Puhó

**Affiliations:** 1 Department of Dermatology, Aarhus University Hospital, Aarhus, Denmark; 2 Department of Rheumatology, Aarhus University Hospital, Aarhus, Denmark; 3 Department of Clinical Epidemiology and Center for Population Medicine, Aarhus University Hospital and Aarhus University, Olof Palmes Allé 43‒45, 8200 Aarhus N, Denmark

**Keywords:** Atopic dermatitis, Venous thromboembolism, Cancer, Risk, Cohort

## Abstract

**Abstract:**

Atopic dermatitis is a risk factor for venous thromboembolism which may be the first manifestation of occult cancer. We examined whether a venous thromboembolism in patients with atopic dermatitis is a marker of occult cancer. We used Danish health registries to conduct this population-based cohort study. Patients with a first-time diagnosis of venous thromboembolism and a history of atopic dermatitis were identified from the Danish National Patients Registry from 1980 through 2022. We calculated the absolute risk of cancer treating death as a competing event. As a measure of relative risk, we calculated standardized incidence ratios (SIRs) for cancer among patients with venous thromboembolism and atopic dermatitis and compared the observed cancer incidence to that of the general Danish population. We identified 582 patients with a first venous thromboembolism diagnosis and a history of atopic dermatitis. During the first year of follow‐up, the absolute risk of overall cancer was 1.7%, corresponding to an SIR of 2.90 (95% confidence interval [CI] 1.39–5.34). The overall SIR decreased to 1.12 (95% CI 0.74–1.62) during the subsequent years of follow‐up. Although the risk estimates were imprecise, an elevated cancer risk following venous thromboembolism in patients with atopic dermatitis cannot be ruled out, particularly within the first year after venous thromboembolism, when compared to the cancer risk in the general population.

**Graphical abstract:**

Atopic dermatitis, venous thromboembolism and cancer: a cohort analysis. VTE, venous thromboembolism; AD, atopic dermatitis; CI, confidence interval

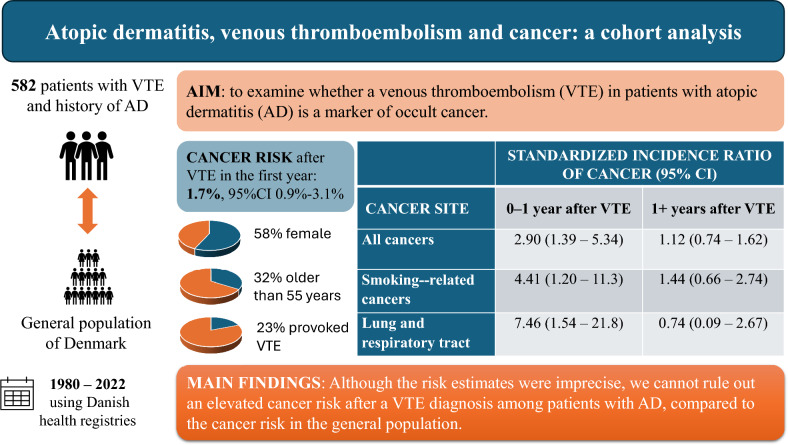

**Supplementary Information:**

The online version contains supplementary material available at 10.1007/s11239-025-03175-2.

## Highlights

- Atopic dermatitis is a risk factor for venous thromboembolism.
- Venous thromboembolism may be the first manifestation of occult cancer.
- It is unclear whether a venous thromboembolism in patients with atopic dermatitis is a marker of occult cancer.
- During the first year of follow‐up, the absolute risk of overall cancer was 1.7% among 582 patients with venous thromboembolism and atopic dermatitis.
- Although the risk estimates were imprecise, an elevated cancer risk following venous thromboembolism in patients with atopic dermatitis cannot be ruled out.

**Supplementary Information:**

The online version contains supplementary material available at 10.1007/s11239-025-03175-2.

## Introduction

Venous thromboembolism (VTE), including deep venous thrombosis (DVT) and pulmonary embolism (PE) [1] is a multifactorial condition with an overall incidence of one to two events per 1,000 person-years [2–4]. However, the incidence is substantially higher in individuals aged 55 years and older (10 VTEs per 1,000 person-years) [5].

An association between cancer and VTE has been known for decades, and robust evidence has documented that VTE may be the first manifestation of occult cancer [6, 7]. Cancer patients have a nine-fold higher VTE risk than the general population [8]. The occurrence of VTE is associated with interruption of cancer treatment, decreased quality of life, and increased morbidity and mortality [9–11].

Atopic dermatitis (AD) is a common chronic inflammatory skin disorder [12]. There is increasing evidence that the inflammation related to AD is systemic and not only restricted to the skin [12]. AD is associated with multiple comorbidities [12], one potentially being VTE. A recent meta-analysis based on six cohort studies showed an increased risk of DVT (pooled hazard ratio 1.15, 95% confidence interval [CI] 1.04–1.27) but not of PE (pooled hazard ratio 0.99, 95% CI 0.87–1.13) in patients with AD [13]. The evidence for an association between AD and cancer risk is less clear [14–16]. A meta-analysis has suggested an elevated risk of keratinocyte carcinoma and kidney cancer as well as lower risk of lung and respiratory system cancers in patients with AD compared to people without AD [17].

VTE in patients with AD may reflect the chronic inflammation associated with AD [13]. However, given the known association between VTE and cancer, VTE in patients with AD could also be a manifestation of occult cancer. Whether the risk of cancer following VTE is elevated in patients with AD has not been investigated. Therefore, we examined the risk of cancer after a diagnosis of VTE in patients with AD and compared the number of observed cancer events among patients with VTE and AD to the expected number of cancer events based on general population rates.

## Methods

### Design and setting

This nationwide population-based cohort study was conducted in Denmark from January 1, 1980, to December 31, 2022. Denmark’s National Health Service offers universal, tax-funded healthcare, guaranteeing free access to hospitals and general practitioners, as well as partial reimbursement for prescribed medications for all Danish residents [18]. Individual-level linkage across all administrative and health registries is possible using a unique personal identification number assigned to residents at birth or upon immigration [18, 19].

The key data sources used in this study are described below:

- *The Danish Civil Registration System*: Established in 1968, this comprehensive registry records individual-level demographic and vital statistics for all residents of Denmark. It provides continuous, real-time updates on key life events, including birth, immigration, emigration, and mortality [19].
- *The Danish National Patient Registry*: The registry has systematically recorded non-psychiatric hospital admissions since 1977. The registry was expanded in 1995 to include outpatient clinic visits, emergency department encounters, and psychiatric contacts [20]. It contains detailed information on dates of admission and discharge, primary and secondary diagnoses, and data on surgical interventions. This registry was used to create the study cohort.
- *The Danish Cancer Registry*: Established in 1943, this nationwide registry records all incident cancer cases in Denmark. It contains detailed tumor-specific data, including topography, morphology, stage at diagnosis, and date of diagnosis [21].

### Study cohort

**Fig. 1:**
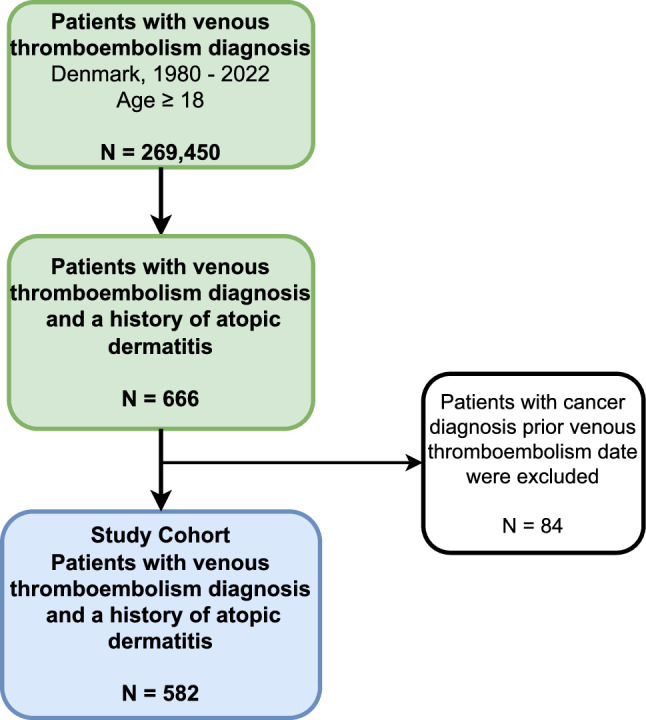
Flowchart illustrating the study cohort

We used the Danish National Patient Registry to identify all patients with a first-time diagnosis of VTE using primary and secondary discharge diagnoses registered during inpatient hospitalizations or outpatient clinic visits between 1980 and 2022. We excluded VTE diagnoses from emergency departments due to their low predictive value [22]. Patients with a cancer diagnosis (except non-melanoma skin cancer) before their date of the first VTE admission were excluded from the cohort. Next, we identified VTE patients who had a recorded diagnosis of AD prior to their VTE diagnosis date (Fig. 1). VTE and AD diagnoses were determined using *International Classification of Diseases* (ICD) codes, with *Eighth Revision* (ICD-8) before 1994 and *Tenth Revision* (ICD-10) thereafter (Table S1). The index date was defined as the date of the first VTE admission.

### Outcomes

Information on incident cancer diagnoses were obtained from the Danish Cancer Registry. All primary cancers were included in the analyses, except non-melanoma skin cancer. Due to the limited number of cancer cases, only smoking-related cancers could be included as separate outcomes. Cancers of the lung and respiratory tract were included in the smoking-related cancer outcome and were also reported as a separate endpoint. For a list of ICD-10 cancer codes used in this study, see Table S1.

### Statistical analyses

The cohort of patients with VTE and a history of AD was described according to sex, age at VTE diagnosis, calendar year of VTE, VTE type (DVT or PE), presence of provoking factors (pregnancy, trauma/fractures or surgery) of VTE within three months prior to their VTE diagnosis, time between AD diagnosis and VTE, and Charlson Comorbidity Index (CCI), presented as numbers with percentages and medians with first and third quartiles. Provoking factors such as estrogen therapy, the puerperium, or immobilization were not included in the definition of provoked VTE.

Patients were followed from the date of VTE to incident cancer, death, emigration, or end of study (31 December 2022), whichever came first. As an absolute risk measure, we calculated cumulative incidence proportions of cancer, treating death as a competing risk. We computed the age-, sex-, and calendar year-standardized incidence ratio (SIR) as a measure of relative risk of cancer diagnosis after VTE using national incidence rates in the general Danish population as the reference. The SIR is an established association measure used to study cancer risk [23], which contrasts the number of observed cancer cases among patients with VTE and a history of AD to the number of cancer cases that would be expected if patients with VTE and AD had had the same cancer risk as the Danish general population. Expected cancer rates were calculated using national incidence data from the Danish Cancer Registry, stratified by sex, age, and calendar period in 5-year intervals. CIs were estimated assuming a Poisson distribution, with exact 95% CIs applied when less than 10 cancers were observed and the Byar approximation used otherwise [24]. The SIRs were calculated for the first year after VTE and the > 1 year follow-up period.

All statistical analyses were performed using the SAS statistical software, Version 9.4 (SAS Institute Inc., Cary, NC, USA). The study followed the STrengthening the Reporting of OBservational studies in Epidemiology (STROBE) guidelines (Table S2).

## Results

We identified 582 patients with a first VTE diagnosis and a history of AD (Fig. 1). Among the VTE patients with AD, 58% were female, 32% were older than 55 years, and 9% had a high comorbidity score (CCI score ≥ 3). 244 patients (42%) had DVT, 169 patients (29%) had PE and 169 (29%) had other VTE. 23% had a provoking factor of VTE within three months prior their index date (Table 1).

**Table 1:** Characteristics of patients with venous thromboembolism and a history of atopic dermatitis, Denmark, 1980–2022

	Patients with venous thromboembolism and a history of atopic dermatitis	
	N	%
*Overall*	582	100
Sex		
Female	336	58
Male	446	42
Age at VTE diagnosis, years (median, Q1–Q3)	44.2 (31.1–59.4)	
Age at VTE diagnosis, years		
18–44 years	299	51
45–54 years	98	17
55–64 years	77	13
65–74 years	61	11
75 + years	47	8
Calendar period of VTE diagnosis		
1980–1987	8	1
1988–1995	19	3
1996–2000	22	4
2001–2005	45	8
2006–2010	98	17
2011–2015	167	29
2016–2022	223	38
VTE type		
Deep venous thrombosis	244	42
Pulmonary embolism	169	29
Other VTE	169	29
Provoked VTE (without cancer as provoking factor)	136	23
Time between AD diagnosis and VTE		
≤ 12 months	28	5
> 12 months	554	95
Charlson Comorbidity Index		
Low	247	42
Medium	284	49
High	51	9
Follow-up time after VTE diagnosis, years (median, Q1–Q3)	5.3 (2.3–10.9)	

VTE, venous thromboembolism; AD, atopic dermatitis

In total, 38 cancers were observed during the study period. During the first year of follow‐up, the absolute risk of overall cancer was 1.7% (95% CI 0.9%-3.1%), corresponding to an SIR of 2.90 (95% CI 1.39–5.34). The overall SIR decreased to 1.12 (95% CI 0.74–1.62) during the subsequent years of follow‐up. The strongest associations were observed for smoking-related cancers (including lung cancer) with an SIR of 4.41 (95% CI 1.20–11.3) during the first year of follow‐up and 1.44 (95% CI 0.66–2.74) during the subsequent years of follow‐up (Table 2). When analyzing cancers of the lung, bronchi, and trachea as a separate endpoint, we observed an SIR of 7.46 (95% CI 1.54–21.8) during the first year of follow‐up. However, the absolute risks were low.

**Table 2:** Standardized incidence ratios of cancers among patients with venous thromboembolism and a history of atopic dermatitis, Denmark 1980–2022

	Observed number of cancer cases	Standardized incidence ratios (95% confidence interval)	
		0–1 year after VTE	1 + years after VTE
*Cancer site*			
All cancers	38	2.90 (1.39–5.34)	1.12 (0.74–1.62)
Smoking-related cancers	13	4.41 (1.20–11.3)	1.44 (0.66–2.74)
Lung, bronchi and trachea	5	7.46 (1.54–21.8)	0.74 (0.09–2.67)

VTE, venous thromboembolism

## Discussion

In this study, we identified 582 patients with VTE after AD, and 38 incident cancers were observed during the study period. Although the risk estimates were imprecise, our findings indicated that the overall cancer risk may be elevated compared to the general population, particularly within the first year after VTE. Notably, the risk of smoking-related cancers within the first year was elevated and was primarily driven by an increased risk of cancers of the lung and respiratory tract.

To the best of our knowledge, no previous studies have examined the risk of cancer after VTE diagnosis among patients with AD. However, an increased cancer risk compared with the general population following VTE has been described in patients with chronic diseases, such as diabetes [25] and liver diseases [26]. In our study, risk estimates were imprecise owing to a small study population and a low absolute risk. However, we did identify an elevated lung cancer risk within the first year after VTE, suggesting the detection of occult cancers during this period or shared risk factors between lung cancer, VTE and AD. For instance, smoking has been suggested as a risk factor for both AD and cancer [27, 28].

The current guidelines suggest that extensive screening for occult cancer in patients with VTE generally leads to a higher rate of cancer detection [29]. However, these strategies may not be translated into improved cancer-related mortality.

The major strength of this study was the use of nationwide population-based registries with complete follow-up, reducing the risk of referral and selection bias. Additionally, the positive predictive values of diagnostic coding in the Danish National Patient Registry for VTE [30] and comorbidities [31], as well as for cancer in the Danish Cancer Registry [21], have been evaluated as generally high. A few limitations should be taken into account when interpreting these findings. First, the potential for detection bias should be considered when examining the association between VTE, AD and subsequent cancer during the first year of follow-up. Second, we controlled for sex, age, and calendar period. However, we were not able to control for smoking, which may increase the risk of developing atopic dermatitis due to the harmful effects of tobacco smoke on the skin barrier and immune system, and smoking is a strong risk factor for cancer [32]. Our findings are consistent with this explanation. Third, the cohort of AD patients was identified through the Danish National Patient Registry, which primarily captures severe cases of AD, as inclusion requires at least one contact with the secondary healthcare system. Moreover, the cohort of AD patients was restricted to hospital in- and outpatients. The registration of outpatient clinic visits in the Danish National Patient Registry started in 1995 [20]. The positive predictive value of the AD diagnosis coding is high [33], but any reduced sensitivity would tend to minimize the strength of the associations we recorded [34]. Fourth, as we compared the cancer risk of patients with both VTE and AD to that of the general population, we cannot determine whether the potentially elevated cancer risk is attributable to VTE, AD, or a combination of both diseases. Finally, the calculations were based on a limited number of cancer diagnoses, which reduced the robustness of the results. Therefore, the estimates should be interpreted with caution. The limited precision impeded detailed analyses of specific cancer outcomes and patient subgroups. As this study focused on VTE patients in Denmark, the generalizability of the findings may be influenced by characteristics specific to the Danish healthcare setting.

Although the risk estimates were imprecise, we cannot rule out an elevated cancer risk after a VTE diagnosis among patients with AD, compared to the cancer risk in the general population. Patients with AD and VTE should follow the same guidelines for occult cancer screening as other patients with VTE until more evidence becomes available.

## Supplementary Information

Below is the link to the electronic supplementary material.

Supplementary file1 (DOCX 28 KB)

## Data Availability

No datasets were generated or analysed during the current study.
