# Supplementary material for: Atopic dermatitis, venous thromboembolism and cancer: a cohort analysis
Source: J Thromb Thrombolysis. 2025 Sep 4;58(8):1120–6. doi: 10.1007/s11239-025-03175-2 (PMC12740962; doi:10.1007/s11239-025-03175-2)
Supplement: Supplementary file 1 — Supplementary file1 (DOCX 28 KB) [file 11239_2025_3175_MOESM1_ESM.docx]

**Atopic dermatitis, venous thromboembolism and cancer: a cohort analysis**

Sissel Brandt Toft Sørensen^1,2^ (ORCID 0000-0002-9243-9736), Cecilia H. Fuglsang^3^ (ORCID 0000-0003-2789-5003), Erzsébet Horváth-Puhó^3^ (ORCID 0000-0002-3594-2212)

^1^ Department of Dermatology, Aarhus University Hospital, Aarhus, Denmark

^2^ Department of Rheumatology, Aarhus University Hospital, Aarhus, Denmark

^3^ Department of Clinical Epidemiology and Center for Population Medicine, Aarhus University Hospital and Aarhus University, Aarhus, Denmark

**Corresponding author**

Erzsébet Horváth-Puhó

Department of Clinical Epidemiology and Center for Population Medicine, Aarhus University Hospital and Aarhus University

Olof Palmes Allé 43‒45, 8200 Aarhus N, Denmark

Tel: +45 87168934

E-mail: ep@clin.au.dk

### **Table S1.** Diagnosis codes used in the study.

|  | **ICD-8 codes** | **ICD-10 codes** | **Type of contact and diagnosis** |
| --- | --- | --- | --- |
| **Atopic dermatitis** | 691 | L20.2, L20.8, L20.9 | Inpatient and outpatient, primary and secondary diagnoses |
| **Venous thromboembolism** |  |  | Inpatient and outpatient, primary and secondary diagnoses |
| Deep vein thrombosis | 451.00 | I80.1–3 |  |
| Pulmonary embolism | 450.99 | I26 |  |
| Other venous thromboembolisms (including superficial venous thrombosis) | 450 (all subcodes, except 450.99), 451 (all subcodes, except 451.00), 452, 453, 671, 673 | I80.0, I81, I82, O88.2, T81.7C, T81.7D |  |
| **All cancers** |  | C00–C96  (excluding C44) | Any cancer diagnosis (except non-melanoma skin cancer), defined as a diagnosis in the Danish Cancer Registry |
|  |  |  |  |
| **Smoking-related cancers** |  |  |  |
| Tongue |  | C01, C02 |  |
| Oral cavity |  | C03, C04, C05, C06 |  |
| Tonsil and oropharynx |  | C09, C10 |  |
| Pancreas |  | C25 |  |
| **Lung, bronchi, and trachea** |  | C33, C34 |  |
| Kidney |  | C64 |  |
| Urinary bladder |  | C67 |  |
| Thyroid |  | C73 |  |

Abbreviations: ICD: International Classification of Diseases, Eight or Tenth Revision

**Table S2.** STROBE checklist

|  | **Item No.** | **STROBE item** | **Location in manuscript where items are reported** |
| --- | --- | --- | --- |
| **Title and abstract** | 1 | (*a*) Indicate the study’s design with a commonly used term in the title or the abstract | Title and Abstract |
|  |  | (*b*) Provide in the abstract an informative and balanced summary of what was done and what was found | Abstract (Paragraphs 2 and 3) |
| **Background/****rationale** | 2 | Explain the scientific background and rationale for the investigation being reported | Introduction (Paragraphs 1-3) |
| **Objectives** | 3 | State specific objectives, including any prespecified hypotheses | Introduction (Paragraph 4) |
| **Study design** | 4 | Present key elements of study design early in the paper | Introduction (Paragraph 4)  Methods (Paragraph 1) |
| **Setting** | 5 | Describe the setting, locations, and relevant dates, including periods of recruitment, exposure, follow-up, and data collection | Methods/Design and setting  Methods/Study cohort  Supplementary material (Table S1) |
| **Participants** | 6 | (*a*) *Cohort study*—Give the eligibility criteria, and the sources and methods of selection of participants. Describe methods of follow-up  *Case-control study*—Give the eligibility criteria, and the sources and methods of case ascertainment and control selection. Give the rationale for the choice of cases and controls  *Cross-sectional study*—Give the eligibility criteria, and the sources and methods of selection of participants  (*b*) *Cohort study*—For matched studies, give matching criteria and number of exposed and unexposed  *Case-control study*—For matched studies, give matching criteria and the number of controls per case | Methods/Study cohort  Methods/Statistical analysis (Paragraph 2)  Results (Paragraph 2) |
| **Variables** | 7 | Clearly define all outcomes, exposures, predictors, potential confounders, and effect modifiers. Give diagnostic criteria, if applicable | Methods/Study cohort, Outcomes, Statistical analysis  Supplementary material (Table S1) |
| **Data sources/** **measurement** | 8 | For each variable of interest, give sources of data and details of methods of assessment (measurement). Describe comparability of assessment methods if there is more than one group | Methods/Design and setting, Study cohort  Supplementary material (Table S1) |
| **Bias** | 9 | Describe any efforts to address potential sources of bias | Methods/ Statistical analyses (Paragraphs 1-2) |
| **Study size** | 10 | Explain how the study size was arrived at | Methods/Study cohort  Results (Paragraph 1) |
| **Quantitative** **variables** | 11 | Explain how quantitative variables were handled in the analyses. If applicable, describe which groupings were chosen and why | Results (Paragraphs 1-2)  Table 1 |
| **Statistical** **methods** | 12 | (*a*) Describe all statistical methods, including those used to control for confounding | Methods/ (under the subheading *Statistical analyses*) |
|  |  | (*b*) Describe any methods used to examine subgroups and interactions | - |
|  |  | (*c*) Explain how missing data were addressed | - |
|  |  | (*d*) *Cohort study*—If applicable, explain how loss to follow-up was addressed  *Case-control study*—If applicable, explain how matching of cases and controls was addressed  *Cross-sectional study*—If applicable, describe analytical methods taking account of sampling strategy | Methods/ Statistical analyses (Paragraph 2) |
|  |  | (*e*) Describe any sensitivity analyses | - |
| **Participants** | 13 | (a) Report numbers of individuals at each stage of study—eg numbers potentially eligible, examined for eligibility, confirmed eligible, included in the study, completing follow-up, and analysed  (b) Give reasons for non-participation at each stage  (c) Consider use of a flow diagram | Results (Paragraph 1)  Figure 1 |
| **Descriptive** **data** | 14 | (a) Give characteristics of study participants (eg demographic, clinical, social) and information on exposures and potential confounders | Results (Paragraphs 1 and 2)  Table 1 |
|  |  | (b) Indicate number of participants with missing data for each variable of interest |  |
|  |  | (c) *Cohort study*—Summarise follow-up time (eg, average and total amount) |  |
| **Outcome data** | 15 | *Cohort study*—Report numbers of outcome events or summary measures over time | Results (Paragraph 2)  Table 2  Supplementary material (Table S1) |
|  |  | *Case-control study—*Report numbers in each exposure category, or summary measures of exposure |  |
|  |  | *Cross-sectional study—*Report numbers of outcome events or summary measures |  |
| **Main results** | 16 | (*a*) Give unadjusted estimates and, if applicable, confounder-adjusted estimates and their precision (eg, 95% confidence interval). Make clear which confounders were adjusted for and why they were included  (*b*) Report category boundaries when continuous variables were categorized  (*c*) If relevant, consider translating estimates of relative risk into absolute risk for a meaningful time period | Results (Paragraph 2)  Table 2 |
| **Other analyses** | 17 | Report other analyses done—eg analyses of subgroups and interactions, and sensitivity analyses | - |
| **Discussion** |  |  |  |
| **Key results** | 18 | Summarise key results with reference to study objectives | Discussion (Paragraphs 1) |
| **Limitations** | 19 | Discuss limitations of the study, taking into account sources of potential bias or imprecision. Discuss both direction and magnitude of any potential bias | Discussion (Paragraph 4) |
| **Interpretation** | 20 | Give a cautious overall interpretation of results considering objectives, limitations, multiplicity of analyses, results from similar studies, and other relevant evidence | Discussion (Paragraphs 2-3) |
| **Generalisability** | 21 | Discuss the generalisability (external validity) of the study results | Discussion (Paragraph 4) |
| **Other information** |  |  |  |
| **Funding** | 22 | Give the source of funding and the role of the funders for the present study and, if applicable, for the original study on which the present article is based | Paragraph *Competing interests and funding* |
